# An Experimental Inquiry into the Nature of Gravelly and Calculous Concretions in the Human Subject; and the Effects of Alkaline and Acid Substances on Them, in and out of the Body

**Published:** 1806-09

**Authors:** Thomas Egan


*An experimental Inquiry into the Nature of Gravelly and Calculous Concretions in the Human Subject; and the Effects of Alkaline and Acid Substances on them, in and out of the Body.* *By* THOMAS EGAN, *M. D. M.R.I.A.*

[ Continued from our last, pp. 153—165. ]

THESE facts being pretty well established and acknowledged, it is time to enquire how far we may account for them: and whether experiments, instituted out of the body, may not throw some light on this subject. Dr. Saunders, in a letter to Dr. Percival, (Percival's Essays, Medical cal and Experimental, vol. iii.) on the subject of carbonic acid as a solvent of calculous concretions, observes, "If a more powerful and active solvent than any hitherto known shall be discovered, it is highly probable that such a discovery can only be made by a rational and chemical inquiry into the powers of different bodies of combining with the contents of the urine, and preserving them in a fluid state out of the body." Now, on the other hand, we may presume, that whatever substances cause a separation or precipitation of uric acid, in an aggregate state, from healthy urine, will give rise to these disorders. For we are not to forget that the uric acid, which forms so large a proportion of calculous concretions, and the entire of the gravelly, is a natural secretion from the blood, performed by the functions of the kidneys, and excreted by the urine, and can only be prejudicial by a previous morbid separation from it within the body. With this necessary view of the subject before us, (for which we are, as already observed, indebted to Boerhaave,) I resolved to try, 1st. What might be the effects of acids of different kinds on healthy urine, as to their influence in causing this same previous precipitation; and, 2dly, that of alkaline substances in preventing it. And here it must be observed, that to draw any satisfactory conclusions from experiments made with these substances out of the body, we must suppose they reach the kidneys and blend with the urine, still possessing their relative distinctive properties; and that this takes place, we have every reason to presume. Drs. Percival and Saunders, Mr. Bewley, and many others, have ascertained the presence of carbonic acid, in an uncombined state, in the urine of those who drank the mephitic water for some days: an acid certainly foreign to its recent healthy state; for, after repeated trials, by heating it to nearly ebullition in one of Priestly's air bottles, I never could procure the separation or transition of a single bubble of carbonic acid into a jar of lime water. And if this weak acid reaches the kidneys undecomposed or uncombined, we shall have less difficulty in believing the more powerful ones may do so. That the tartarous acid in the combination of the acidulous tartarite of potash exerts powerful effects on the functions of the kidneys, is well known; and that the urine is at the same time rendered more acid, I have repeatedly ascertained by the usual tests.

We may say the same of the other vegetable acids, which manifest also diuretic powers, and increase the natural acidity of the urine. Linnæus, in his second volume of the the *Amœnitates Academicæ, De Genesi Calculi,* already quoted, mentions his having made the following experiments to this purpose. He says: "Hisce diebus ipse experimentum institui cum urina; hæc communiter a solutione lacmus parum admodum rufescit; at si libram unam vel alteram vini Rhenani, vel alterius vini acidi hauserim, post horam unam vel plures, valde rubra et rutilans evadit urina, ab affusa solutione lacmus; certo indicio, acidum vini totum corpus permeasse, et urinam infecisse." Nor should we wonder that these energetic substances should pass unaltered to the kidneys, when we find so many mild vegetable matters do so. I will not mention the communication of so volatile a principle as odour, but will more particularly dwell on that of colour. Rhubarb, turmeric, madder, and many other substances, so completely impart their colour to urine, that they would appear to be very little altered. Nay, the juice of the beta vulgaris, a mild esculent of the pentandrous class, so deeply reddens it as to cause it to be mistaken for bloody urine, of which a late instance has occurred in my practice.

As to alkaline substances, it has been at all times known that they communicate their properties to this excrementitious liquor. A perseverance in the use of the *aqua kali puri* of the shops for a few days, even in small doses, converts its acescent into the alkaline state; and we have every reason to suppose that the same takes place with the carbonates, which are taken in so much larger quantities. This seems confirmed by experiments made in London and Paris; and the alkalescent impregnation of the urine was ascertained by the formation and precipitation of the acidulous tartarite of potash upon the addition of the tartarous acid. Yet, from a good deal of experience in these matters, I may aver, that as to the carbonates the dose must be considerable, (which was the case in London,) and continued for some time, having frequently given two scruples of desiccated soda (containing, according to Kirwan, 23-94 grains,) in the twenty-four hours, for some days together, without any diminution of the usual acidity of this liquor.

For the information of such of my readers as may not be of the medical profession, I must here observe, that physicians distinguish two kinds of urine: the one rendered immediately after meals, and much diluted, before the process of digestion, or state of sleep, can take place; always more or less limpid; being comparatively less charged with the natural component parts of urine, (the urée, or extractive extractive colouring matter, in particular,) and called *urina potus*, to distinguish it from the *urina sanguinis*; rendered many hours after meals and sleep, the taking no more than a necessary quantity of liquids, and containing the usual proportion of saline and other ingredients; more especially the urée, to which it owes its natural citrine colour.

This last, therefore, was that employed in the following experiments, if not otherwise specified; with the chemical history of which I must suppose gentlemen of the profession now tolerably well acquainted, being so fully and accurately detailed in the tenth volume of the *Connoissances Chimiques*.

Having, in the preceding pages, insisted so much on the acid and acescent drinks as occasional causes of these complaints, the first object seemed to be, to ascertain whether the urine of those most subject to them, or actually labouring under them, was more relatively acid. We have already seen, from a register of these patients, kept for forty years in the hospital of Luneville, that the early of life, from two to six years of age inclusive, is most liable to calculous affections. Now, the urine of healthy chilbren is always found more acid than that of adults, generally in the proportion of two to one. Whilst several drops of the latter are requisite to redden a given quantity of infusion of litmus, a single drop of the former turns it to a clear red. Paper stained with an infusion of turmeric, and reddened by an alkali, was immediately restored to its colour by a single emersion in the urine of children; an effect which required some time in that of adults. And that this should be the case we shall not be so much surprised at, when we consider the nature of their diet; and that, in addition to the phosphoric and uric, their urine contains the benzoic acid in considerable quantity, the proportion of which is found afterwards progressively to diminish with their advancement in life.

The constant opportunity I have of attending to those subjects, enables me to say, that the urine of gravelly patients, when fresh rendered, nay, after standing many hours, in a temperature of sixty degrees, is relatively more acid than the healthy, sometimes as much so as the gouty; and frequently continues so, even after depositing its gravelly matter. An exception to this, however, sometimes occurs in gouty habits; their urine depositing copiously this acid substance, and yet manifesting no increase, but sometimes rather decreased, acescency; for with them a considerable considerable diminution of the quantity of the usually excreted superacidulated phosphoric salt often takes place, as shall be fully explained upon another occasion.

Having premised these observations, it is now time to consider what effects acid substances are productive of, when mixed, out of the body, with this very complicated liquor. And here, to prevent repetition, I will observe, that that generally used was rendered fresh in the morning, in the quantity from three to four ounces, (unless otherwise specified,) being that most easily retained at one time in the bladder. The quantity of acid extremely small, for obvious reasons, and seldom increasing its acescent properties (as ascertained by the usual tests) beyond what frequently occurs in the urine of those who use acescent drinks, or are afflicted with gout or gravel. A standard quantity was always laid by for comparison; and the temperature from sixty to seventy-five degrees, being in autumn 1799. And to begin with the vegetable acids.

Exp. 1.—To four ounces of the urine of an adult was added one drachm of common acetous acid, which, like every other acid, caused no immediate change in it; but in a very short time, and before it cooled down to the temperature of the atmosphere, some extremely minute shining spiculæ, observable only by a lens, were seen floating in it: these gradually increased in number and size, began to reflect the light, and, from being perfectly transparent, soon became coloured, to settle upon the usual cloud, or *nubecula*; which now began to form, adhere to the sides of the glass, and partly fall to the bottom in the shape of small bright red crystals. In the standard, after twelve hours, nothing more observable than the usual *nubecula*; nor was there any sign of crystallization, or separation of uric acid, even after twenty-four.

Exp. 2.—To the same quantity of adult urine were added one drachm and a half of acetous acid, which caused a more copious separation and crystallization of this substance with the foregoing appearances. None observable in the standard after twenty-four hours.

Exp. 3.—To four ounces of urine of a healthy child, who never was observed to pass gravel, and of the usual degree of acidity, was added one drachm of acetous acid, which soon caused an evident and copious separation of crystallized uric acid. The crystals were, however, not quite so coloured; the urine of children not being so much impregnated with the urée, or colouring matter. No such appearance in the standard after twelve hours or more.

 Exp.

Exp. 4.—To four ounces of adult urine, rendered very soon after a tea breakfast, and nearly in a state of *urinæ potus*, was added one drachm of acetous acid. After three hours, a crystallization of minute sandy particles took place. None in the standard even after three days.

Exp. 5.—Thirty drops only, of acetous acid, were added to four ounces of the urine of a gouty patient, æt. 60, and who sometimes felt some slight gravelly tendency. A very copious precipitation of this matter quickly took place. Some observable in the standard, also, the next day.

Exp. 6.—To three ounces of healthy adult urine were added a few drops only of citric acid. A distinct crystallization, but extremely minute, took place. No appearance of any in the standard after many hours. The experiment was repeated with one drachm of filtered citric acid, which only hastened the separation and increased the quantity of crystalline matter.

Finding, by these experiments, and numberless others, with a detail of which it would be unnecessary to take up the time of the academy, that the acetous and citric acids, blended with the urine, separated its uric acid in a crystallized state, I thought it might be interesting to investigate what the effect of the tartarous acid might be, being that which, in an uncombined and partly combined state of acidule, as in the acidulous tartarite of potash, chiefly prevails in the wines and beverage of those countries most subject to these complaints.

Exp. 7.—To four ounces of healthy adult urine were added some drops only of pure tartarous acid. To the same quantity one drachm of acetous acid, which brought them nearly to the same standard of acidity; a circumstance always attended to in the comparative trials with different acids. In that with the tartarous acid the crystals were not only larger and darker coloured, but exceeded in quantity any thing before observed. In that with the acetous acid, a much smaller proportion of minute crystals took place.

Exp. 8.—To four ounces of urine were added two drachms of a filtered solution of acidulous tartarite of potash of the temperature 55 degrees. The usual separation and crystallization took place in large proportion: the crystals, however, much smaller, and less coloured, than those with the uncombined tartarous acid. The two last experiments, frequently repeated, presented the same results.

Exp. 9.—The result of the above experiments having led to some doubt as to the good effects of the carbonic acid acid gas, so much, at one time, recommended by Doctors Percival and Saunders, previous to its more modern alkaline combination in our mephitic as well as super-aërated soda waters:

Into the middle part of Nooth's apparatus were introduced four pounds of fresh rendered healthy urine, and exposed to a stream of carbonic acid gas. After a few hours a copious and beautiful precipitation of uric crystals took place, (notwithstanding the constant agitation from the transmission of the gaseous bubbles,) larger than any I before observed, that from the tartarous acid excepted. In a standard quantity, no distinct crystallization, even after two days. A repetition of the same experiment afforded similar results.

Exp. 10.—Finding the carbonic acid gas productive of similar effects with the other acids hitherto examined, it was natural to inquire how far its combination with the portion of alkaline matter contained in our mephitic and soda waters, so highly surcharged with it, may prevent a separation of this uric acid.

Half an ounce only of the common soda water of the shops, prepared by Mr. Kinsley, was added to four ounces of healthy urine. A similar quantity was impregnated with carbonic acid gas. In the former, after forty-eight hours, or more, no more than the usual nubecula; nor could a single crystal be discovered even by a magnifier. In the latter, an early, copious, and beautiful crystallization. On the result of this experiment, frequently repeated, with various proportions of the mephitic alkaline water, I shall afterwards have occasion to make some remarks.

Though the mineral acids, in an uncombined state, enter not into the matter of our diet, and are no longer considered as lithontriptics, since the notion of the earthy nature of these concretions has been abandoned; yet, as they are sometimes prescribed with other indications, I thought fit to extend my researches, though in a summary way, to them also.

Exp. 11.—To sixteen ounces of urine were added eight drops of very dilute sulphuric acid. To a similar quantity, two scruples of citric acid, to bring them to nearly the same standard of acidity. After a very short interval, in that with citric acid, the usual appearances of transparent floating moleculæ reflecting light, and gradually becoming larger, were observed, and began to adhere to the glass; whilst in the other, after five hours, no such appearances took place. Yet, after forty-eight, here also a precipitation took place of smaller crystals, and less in quantity; for, being collected on a filter, and carefully dried, they weighed only two grains, whilst the former amounted to three. And this is nearly the largest proportion I ever found the above quantity of healthy urine to contain.

Exp. 12.—As the nitrous acid is one of the most active solvents of this matter out of the body, I was curious to ascertain, whether, in the very dilute state in which it must reach the kidneys and bladder, (where its action must have been facilitated by the actual state of solution of this substance,) it would manifest its powers in preventing its separation.

To three ounces of urine, rendered a few hours after breakfast, and, of course, scarcely acid, were added five drops of weak nitrous acid, which did not seem to add very materially to its acescent properties.

To a similar quantity were added four scruples of acetous acid. In less than an hour the former deposited a distinct quantity of gravelly matter in considerable proportion. This, perhaps, we should not be surprised at, when we consider how the action of this acid in that fluid may be determined by superior affinity. In the latter the separation did not take place for a considerable time after. We see, then, that the nitrous acid speedily and powerfully precipitates this acid substance.

Exp. 13.—To six ounces of urine, shewing a strong acescent quality, were added only three drops of strong marine acid. A cloudiness and transparent granular precipitation took place, followed by the formation of extremely minute gravelly concretions, which, even after two days standing, did not assume so red a tinge as that with vegetable acids. This may probably depend upon some action of this acid upon the ureé, or colouring matter; but as to the smallness of the crystals, that evidently depends upon the more speedy precipitation, throwing them down before they can assume their natural size, and leaving but a shade of difference between the crystalline and pulverulent deposits.

Exp. 14.—From the above, then, we are satisfied that the vegetable and mineral acids cause a premature separation and crystallization of the lithic contents of recent healthy urine; but it may be observed that this only takes place under circumstances not at all applicable to the living system, viz. a much inferior temperature, and, in some instances, a contact with the atmospheric air; two powerful powerful promoting causes of crystallization in general, but more especially of the less soluble salts. To determine therefore, this most essential point:

To six ounces of cold but recent urine (in a well-closed phial) were added five drops of very dilute nitrous acid, which were placed on a sand-bath; temperature varying from 80 to about 100 degrees at most. The same quantity, with similar precautions, but without addition, was laid aside in the laboratory as a standard; temperature 56 degrees. After a very short interval indeed, and almost as soon as the urine acquired the temperature of between 80 and 90 degrees, small shining granular particles were observable with a magnifier, began gradually to settle upon a broken kind of nubecula or rather nubeculæ, and to acquire colour and size, though carried up and down the liquor, which was in constant agitation. This experiment again twice latterly repeated, and always with the same result, care being taken to keep the temperature, as nearly as possible, for a few hours, between 90 and 100 degrees,) afforded one of the most pleasing objects imaginable, viz. the formation of this crystalline matter, under all the disadvantages of elevated temperature and constant agitation, from (I may almost say) their primordial moleculæ to the accomplishment of their full size. And here, indeed, they are most beautiful, and not to be distinguished from those spontaneously deposited.

(To be continued.)

